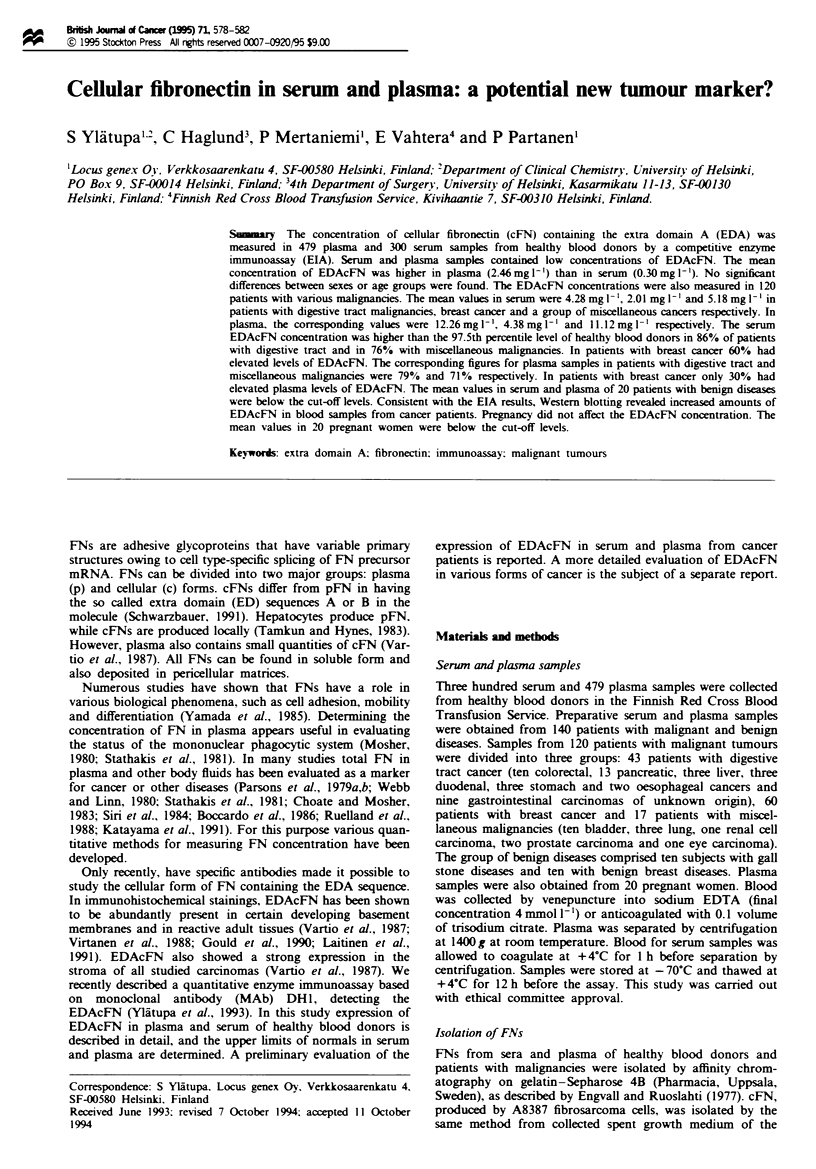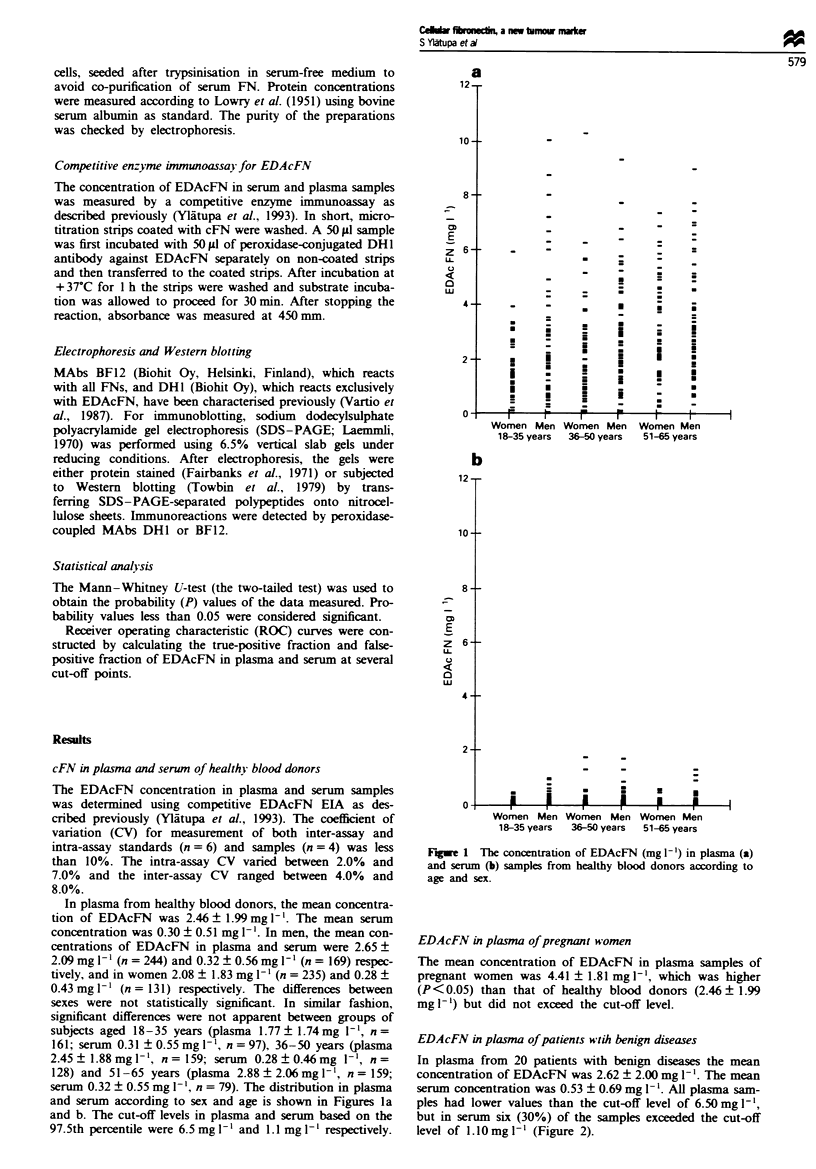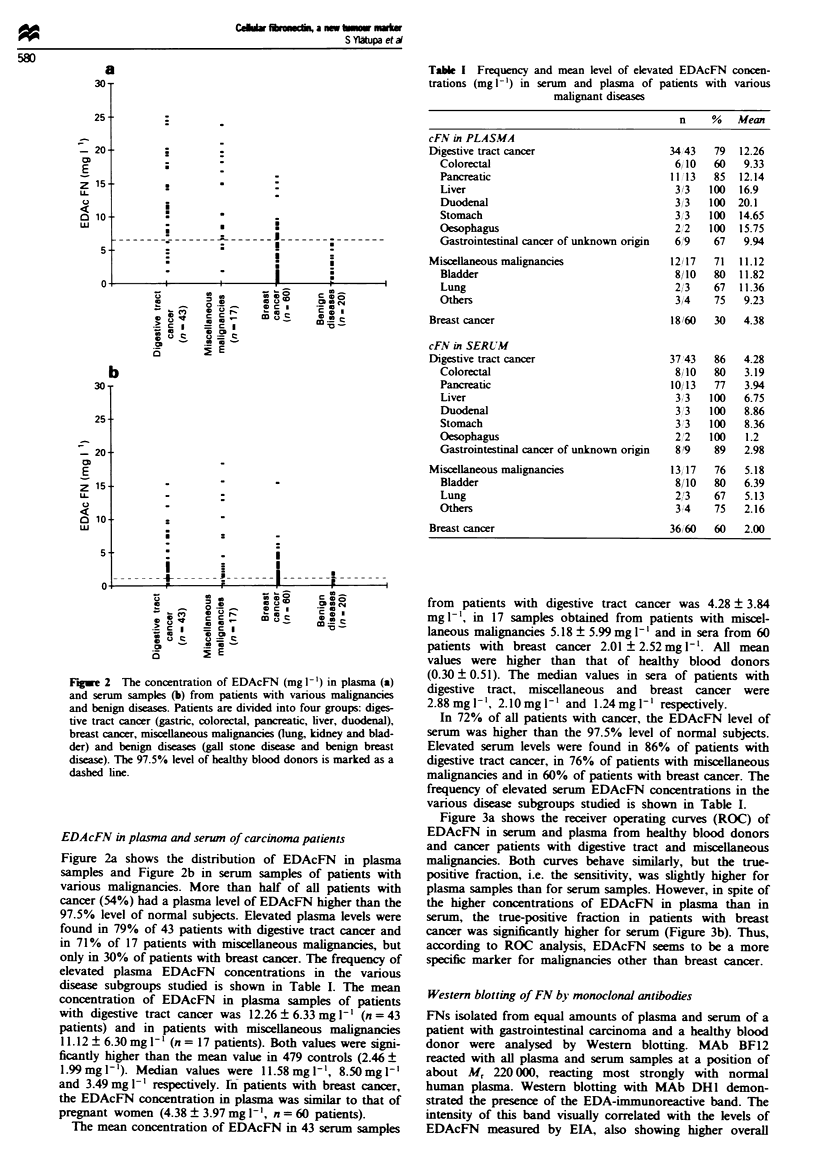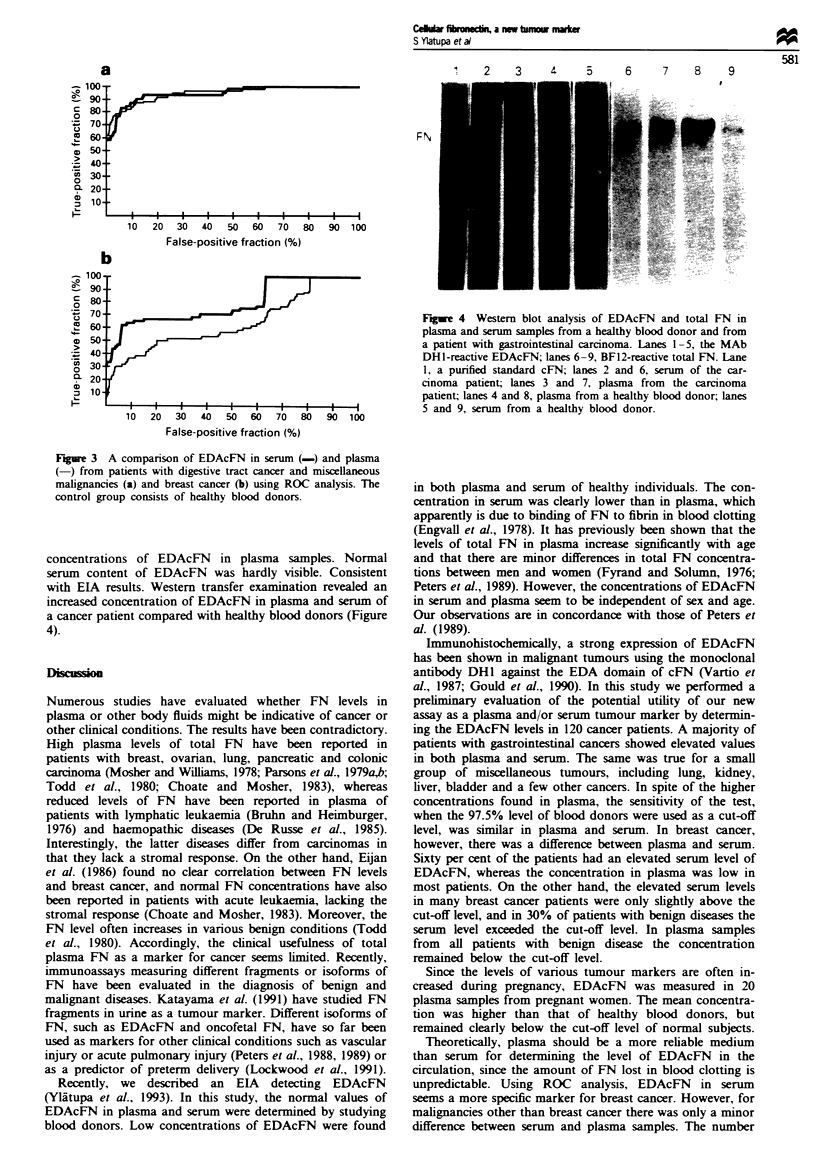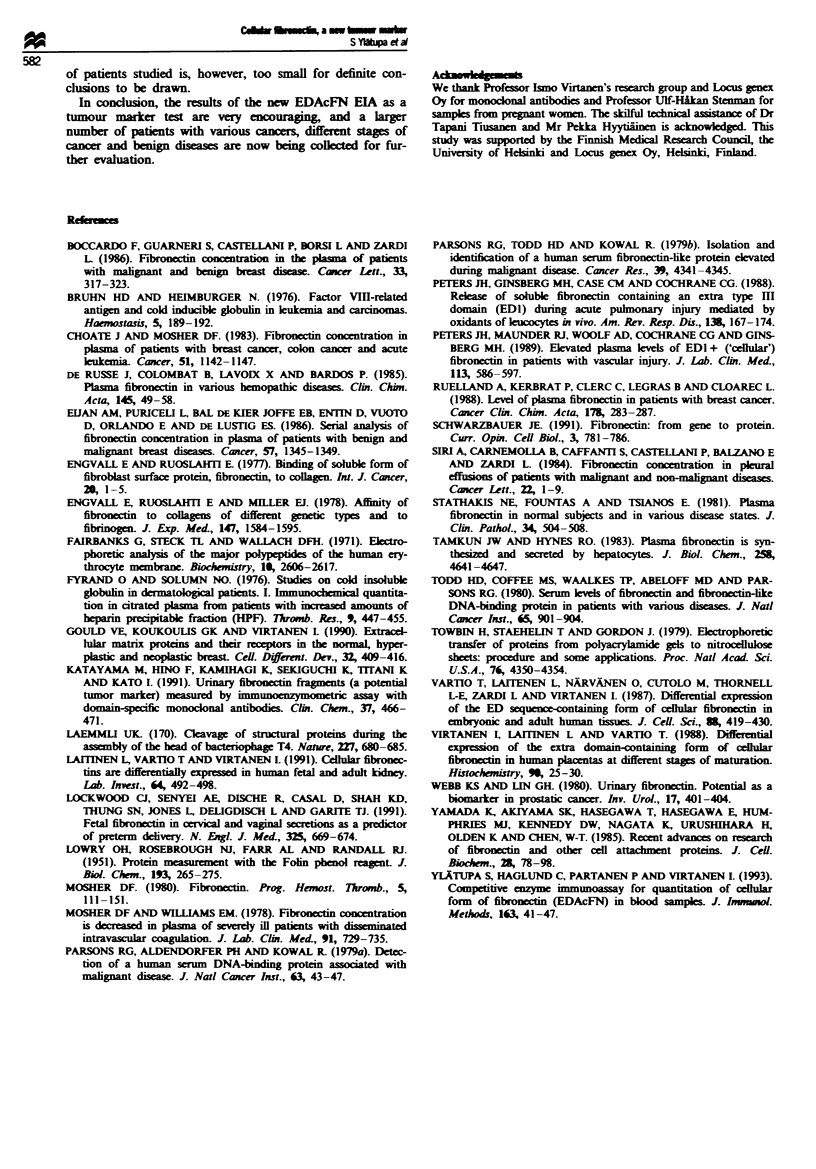# Cellular fibronectin in serum and plasma: a potential new tumour marker?

**DOI:** 10.1038/bjc.1995.112

**Published:** 1995-03

**Authors:** S. Ylätupa, C. Haglund, P. Mertaniemi, E. Vahtera, P. Partanen

**Affiliations:** Locus genex Oy, Helsinki, Finland.

## Abstract

**Images:**


British Journal of Cancer (1995) 71, 578–582

© 1995 Stockton Press All rights reserved 0007–0920/95 $9.00

### Cellular fibronectin in serum and plasma: a potential new tumour marker?

S Ylätupa[1,2], C Haglund[3], P Mertaniemi[1], E Vahtera[4] and P Partanen[1]

[1]*Locus genex Oy, Verkkosaarenkatu 4, SF-00580 Helsinki, Finland;* [2]*Department of Clinical Chemistry, University of Helsinki, PO Box 9, SF-00014 Helsinki, Finland;* [3]*4th Department of Surgery, University of Helsinki, Kasarmikatu 11-13, SF-00130 Helsinki, Finland;* [4]*Finnish Red Cross Blood Transfusion Service, Kivihaantie 7, SF-00310 Helsinki, Finland.*

**Summary** The concentration of cellular fibronectin (cFN) containing the extra domain A (EDA) was measured in 479 plasma and 300 serum samples from healthy blood donors by a competitive enzyme immunoassay (EIA). Serum and plasma samples contained low concentrations of EDAcFN. The mean concentration of EDAcFN was higher in plasma (2.46 mg $l^{-1}$) than in serum (0.30 mg $l^{-1}$). No significant differences between sexes or age groups were found. The EDAcFN concentrations were also measured in 120 patients with various malignancies. The mean values in serum were 4.28 mg $l^{-1}$, 2.01 mg $l^{-1}$ and 5.18 mg $l^{-1}$ in patients with digestive tract malignancies, breast cancer and a group of miscellaneous cancers respectively. In plasma, the corresponding values were 12.26 mg $l^{-1}$, 4.38 mg $l^{-1}$ and 11.12 mg $l^{-1}$ respectively. The serum EDAcFN concentration was higher than the 97.5th percentile level of healthy blood donors in 86% of patients with digestive tract and in 76% with miscellaneous malignancies. In patients with breast cancer 60% had elevated levels of EDAcFN. The corresponding figures for plasma samples in patients with digestive tract and miscellaneous malignancies were 79% and 71% respectively. In patients with breast cancer only 30% had elevated plasma levels of EDAcFN. The mean values in serum and plasma of 20 patients with benign diseases were below the cut-off levels. Consistent with the EIA results, Western blotting revealed increased amounts of EDAcFN in blood samples from cancer patients. Pregnancy did not affect the EDAcFN concentration. The mean values in 20 pregnant women were below the cut-off levels.

**Keywords**: extra domain A; fibronectin; immunoassay; malignant tumours

FNs are adhesive glycoproteins that have variable primary structures owing to cell type-specific splicing of FN precursor mRNA. FNs can be divided into two major groups: plasma (p) and cellular (c) forms. cFNs differ from pFN in having the so called extra domain (ED) sequences A or B in the molecule (Schwarzbauer, 1991). Hepatocytes produce pFN, while cFNs are produced locally (Tamkun and Hynes, 1983). However, plasma also contains small quantities of cFN (Vartio *et al.*, 1987). All FNs can be found in soluble form and also deposited in pericellular matrices.

Numerous studies have shown that FNs have a role in various biological phenomena, such as cell adhesion, mobility and differentiation (Yamada *et al.*, 1985). Determining the concentration of FN in plasma appears useful in evaluating the status of the mononuclear phagocytic system (Mosher, 1980; Stathakis *et al.*, 1981). In many studies total FN in plasma and other body fluids has been evaluated as a marker for cancer or other diseases (Parsons *et al.*, 1979*a*,*b*; Webb and Linn, 1980; Stathakis *et al.*, 1981; Choate and Mosher, 1983; Siri *et al.*, 1984; Boccardo *et al.*, 1986; Ruelland *et al.*, 1988; Katayama *et al.*, 1991). For this purpose various quantitative methods for measuring FN concentration have been developed.

Only recently, have specific antibodies made it possible to study the cellular form of FN containing the EDA sequence. In immunohistochemical stainings, EDAcFN has been shown to be abundantly present in certain developing basement membranes and in reactive adult tissues (Vartio *et al.*, 1987; Virtanen *et al.*, 1988; Gould *et al.*, 1990; Laitinen *et al.*, 1991). EDAcFN also showed a strong expression in the stroma of all studied carcinomas (Vartio *et al.*, 1987). We recently described a quantitative enzyme immunoassay based on monoclonal antibody (MAb) DH1, detecting the EDAcFN (Ylätupa *et al.*, 1993). In this study expression of EDAcFN in plasma and serum of healthy blood donors is described in detail, and the upper limits of normals in serum and plasma are determined. A preliminary evaluation of the expression of EDAcFN in serum and plasma from cancer patients is reported. A more detailed evaluation of EDAcFN in various forms of cancer is the subject of a separate report.

Correspondence: S Ylätupa, Locus genex Oy, Verkkosaarenkatu 4, SF-00580 Helsinki, Finland
Received June 1993; revised 7 October 1994; accepted 11 October 1994

### Materials and methods

### *Serum and plasma samples*

Three hundred serum and 479 plasma samples were collected from healthy blood donors in the Finnish Red Cross Blood Transfusion Service. Preparative serum and plasma samples were obtained from 140 patients with malignant and benign diseases. Samples from 120 patients with malignant tumours were divided into three groups: 43 patients with digestive tract cancer (ten colorectal, 13 pancreatic, three liver, three duodenal, three stomach and two oesophageal cancers and nine gastrointestinal carcinomas of unknown origin), 60 patients with breast cancer and 17 patients with miscellaneous malignancies (ten bladder, three lung, one renal cell carcinoma, two prostate carcinoma and one eye carcinoma). The group of benign diseases comprised ten subjects with gall stone diseases and ten with benign breast diseases. Plasma samples were also obtained from 20 pregnant women. Blood was collected by venepuncture into sodium EDTA (final concentration 4 mmol $l^{-1}$) or anticoagulated with 0.1 volume of trisodium citrate. Plasma was separated by centrifugation at 1400 ***g*** at room temperature. Blood for serum samples was allowed to coagulate at +4°C for 1 h before separation by centrifugation. Samples were stored at −70°C and thawed at +4°C for 12 h before the assay. This study was carried out with ethical committee approval.

### *Isolation of FNs*

FNs from sera and plasma of healthy blood donors and patients with malignancies were isolated by affinity chromatography on gelatin–Sepharose 4B (Pharmacia, Uppsala, Sweden), as described by Engvall and Ruoslahti (1977). cFN, produced by A8387 fibrosarcoma cells, was isolated by the same method from collected spent growth medium of the cells, seeded after trypsinisation in serum-free medium to avoid co-purification of serum FN. Protein concentrations were measured according to Lowry *et al.* (1951) using bovine serum albumin as standard. The purity of the preparations was checked by electrophoresis.

### *Competitive enzyme immunoassay for EDAcFN*

The concentration of EDAcFN in serum and plasma samples was measured by a competitive enzyme immunoassay as described previously (Ylätupa *et al.*, 1993). In short, microtitration strips coated with cFN were washed. A 50 µl sample was first incubated with 50 µl of peroxidase-conjugated DH1 antibody against EDAcFN separately on non-coated strips and then transferred to the coated strips. After incubation at +37°C for 1 h the strips were washed and substrate incubation was allowed to proceed for 30 min. After stopping the reaction, absorbance was measured at 450 mm.

### *Electrophoresis and Western blotting*

MAbs BF12 (Biohit Oy, Helsinki, Finland), which reacts with all FNs, and DH1 (Biohit Oy), which reacts exclusively with EDAcFN, have been characterised previously (Vartio *et al.*, 1987). For immunoblotting, sodium dodecylsulphate polyacrylamide gel electrophoresis (SDS-PAGE; Laemmli, 1970) was performed using 6.5% vertical slab gels under reducing conditions. After electrophoresis, the gels were either protein stained (Fairbanks *et al.*, 1971) or subjected to Western blotting (Towbin *et al.*, 1979) by transferring SDS-PAGE-separated polypeptides onto nitrocellulose sheets. Immunoreactions were detected by peroxidase-coupled MAbs DH1 or BF12.

### *Statistical analysis*

The Mann-Whitney *U*-test (the two-tailed test) was used to obtain the probability (*P*) values of the data measured. Probability values less than 0.05 were considered significant.

Receiver operating characteristic (ROC) curves were constructed by calculating the true-positive fraction and false-positive fraction of EDAcFN in plasma and serum at several cut-off points.

### Results

### *cFN in plasma and serum of healthy blood donors*

The EDAcFN concentration in plasma and serum samples was determined using competitive EDAcFN EIA as described previously (Ylätupa *et al.*, 1993). The coefficient of variation (CV) for measurement of both inter-assay and intra-assay standards ($n = 6$) and samples ($n = 4$) was less than 10%. The intra-assay CV varied between 2.0% and 7.0% and the inter-assay CV ranged between 4.0% and 8.0%.

In plasma from healthy blood donors, the mean concentration of EDAcFN was 2.46 ± 1.99 mg $l^{-1}$. The mean serum concentration was 0.30 ± 0.51 mg $l^{-1}$. In men, the mean concentrations of EDAcFN in plasma and serum were 2.65 ± 2.09 mg $l^{-1}$ ($n = 244$) and 0.32 ± 0.56 mg $l^{-1}$ ($n = 169$) respectively, and in women 2.08 ± 1.83 mg $l^{-1}$ ($n = 235$) and 0.28 ± 0.43 mg $l^{-1}$ ($n = 131$) respectively. The differences between sexes were not statistically significant. In similar fashion, significant differences were not apparent between groups of subjects aged 18-35 years (plasma 1.77 ± 1.74 mg $l^{-1}$, $n = 161$; serum 0.31 ± 0.55 mg $l^{-1}$, $n = 97$), 36-50 years (plasma 2.45 ± 1.88 mg $l^{-1}$, $n = 159$; serum 0.28 ± 0.46 mg $l^{-1}$, $n = 128$) and 51-65 years (plasma 2.88 ± 2.06 mg $l^{-1}$, $n = 159$; serum 0.32 ± 0.55 mg $l^{-1}$, $n = 79$). The distribution in plasma and serum according to sex and age is shown in Figures 1a and b. The cut-off levels in plasma and serum based on the 97.5th percentile were 6.5 mg $l^{-1}$ and 1.1 mg $l^{-1}$ respectively.

**Figure 1** The concentration of EDAcFN (mg $l^{-1}$) in plasma (**a**) and serum (**b**) samples from healthy blood donors according to age and sex.

### *EDAcFN in plasma of pregnant women*

The mean concentration of EDAcFN in plasma samples of pregnant women was 4.41 ± 1.81 mg $l^{-1}$, which was higher ($P < 0.05$) than that of healthy blood donors (2.46 ± 1.99 mg $l^{-1}$) but did not exceed the cut-off level.

### *EDAcFN in plasma of patients wtih benign diseases*

In plasma from 20 patients with benign diseases the mean concentration of EDAcFN was 2.62 ± 2.00 mg $l^{-1}$. The mean serum concentration was 0.53 ± 0.69 mg $l^{-1}$. All plasma samples had lower values than the cut-off level of 6.50 mg $l^{-1}$, but in serum six (30%) of the samples exceeded the cut-off level of 1.10 mg $l^{-1}$ (Figure 2).

**Figure 2** The concentration of EDAcFN (mg $l^{-1}$) in plasma (**a**) and serum samples (**b**) from patients with various malignancies and benign diseases. Patients are divided into four groups: digestive tract cancer (gastric, colorectal, pancreatic, liver, duodenal), breast cancer, miscellaneous malignancies (lung, kidney and bladder) and benign diseases (gall stone disease and benign breast disease). The 97.5% level of healthy blood donors is marked as a dashed line.

*EDAcFN in plasma and serum of carcinoma patients*

Figure 2a shows the distribution of EDAcFN in plasma samples and Figure 2b in serum samples of patients with various malignancies. More than half of all patients with cancer (54%) had a plasma level of EDAcFN higher than the 97.5% level of normal subjects. Elevated plasma levels were found in 79% of 43 patients with digestive tract cancer and in 71% of 17 patients with miscellaneous malignancies, but only in 30% of patients with breast cancer. The frequency of elevated plasma EDAcFN concentrations in the various disease subgroups studied is shown in Table I. The mean concentration of EDAcFN in plasma samples of patients with digestive tract cancer was $12.26 \pm 6.33$ mg $l^{-1}$ ($n = 43$ patients) and in patients with miscellaneous malignancies $11.12 \pm 6.30$ mg $l^{-1}$ ($n = 17$ patients). Both values were significantly higher than the mean value in 479 controls ($2.46 \pm 1.99$ mg $l^{-1}$). Median values were 11.58 mg $l^{-1}$, 8.50 mg $l^{-1}$ and 3.49 mg $l^{-1}$ respectively. In patients with breast cancer, the EDAcFN concentration in plasma was similar to that of pregnant women ($4.38 \pm 3.97$ mg $l^{-1}$, $n = 60$ patients).

The mean concentration of EDAcFN in 43 serum samples from patients with digestive tract cancer was $4.28 \pm 3.84$ mg $l^{-1}$, in 17 samples obtained from patients with miscellaneous malignancies $5.18 \pm 5.99$ mg $l^{-1}$ and in sera from 60 patients with breast cancer $2.01 \pm 2.52$ mg $l^{-1}$. All mean values were higher than that of healthy blood donors ($0.30 \pm 0.51$). The median values in sera of patients with digestive tract, miscellaneous and breast cancer were 2.88 mg $l^{-1}$, 2.10 mg $l^{-1}$ and 1.24 mg $l^{-1}$ respectively.

In 72% of all patients with cancer, the EDAcFN level of serum was higher than the 97.5% level of normal subjects. Elevated serum levels were found in 86% of patients with digestive tract cancer, in 76% of patients with miscellaneous malignancies and in 60% of patients with breast cancer. The frequency of elevated serum EDAcFN concentrations in the various disease subgroups studied is shown in Table I.

Figure 3a shows the receiver operating curves (ROC) of EDAcFN in serum and plasma from healthy blood donors and cancer patients with digestive tract and miscellaneous malignancies. Both curves behave similarly, but the true-positive fraction, i.e. the sensitivity, was slightly higher for plasma samples than for serum samples. However, in spite of the higher concentrations of EDAcFN in plasma than in serum, the true-positive fraction in patients with breast cancer was significantly higher for serum (Figure 3b). Thus, according to ROC analysis, EDAcFN seems to be a more specific marker for malignancies other than breast cancer.

**Table I** Frequency and mean level of elevated EDAcFN concentrations (mg $l^{-1}$) in serum and plasma of patients with various malignant diseases

| | n | % | *Mean* |
|---|---|---|---|
| *cFN in PLASMA* | | | |
| Digestive tract cancer | 34/43 | 79 | 12.26 |
| Colorectal | 6/10 | 60 | 9.33 |
| Pancreatic | 11/13 | 85 | 12.14 |
| Liver | 3/3 | 100 | 16.9 |
| Duodenal | 3/3 | 100 | 20.1 |
| Stomach | 3/3 | 100 | 14.65 |
| Oesophagus | 2/2 | 100 | 15.75 |
| Gastrointestinal cancer of unknown origin | 6/9 | 67 | 9.94 |
| Miscellaneous malignancies | 12/17 | 71 | 11.12 |
| Bladder | 8/10 | 80 | 11.82 |
| Lung | 2/3 | 67 | 11.36 |
| Others | 3/4 | 75 | 9.23 |
| Breast cancer | 18/60 | 30 | 4.38 |
| *cFN in SERUM* | | | |
| Digestive tract cancer | 37/43 | 86 | 4.28 |
| Colorectal | 8/10 | 80 | 3.19 |
| Pancreatic | 10/13 | 77 | 3.94 |
| Liver | 3/3 | 100 | 6.75 |
| Duodenal | 3/3 | 100 | 8.86 |
| Stomach | 3/3 | 100 | 8.36 |
| Oesophagus | 2/2 | 100 | 1.2 |
| Gastrointestinal cancer of unknown origin | 8/9 | 89 | 2.98 |
| Miscellaneous malignancies | 13/17 | 76 | 5.18 |
| Bladder | 8/10 | 80 | 6.39 |
| Lung | 2/3 | 67 | 5.13 |
| Others | 3/4 | 75 | 2.16 |
| Breast cancer | 36/60 | 60 | 2.00 |

*Western blotting of FN by monoclonal antibodies*

FNs isolated from equal amounts of plasma and serum of a patient with gastrointestinal carcinoma and a healthy blood donor were analysed by Western blotting. MAb BF12 reacted with all plasma and serum samples at a position of about $M_r$ 220 000, reacting most strongly with normal human plasma. Western blotting with MAb DH1 demonstrated the presence of the EDA-immunoreactive band. The intensity of this band visually correlated with the levels of EDAcFN measured by EIA, also showing higher overall

**Figure 3** A comparison of EDAcFN in serum (▬) and plasma (—) from patients with digestive tract cancer and miscellaneous malignancies (**a**) and breast cancer (**b**) using ROC analysis. The control group consists of healthy blood donors.

**Figure 4** Western blot analysis of EDAcFN and total FN in plasma and serum samples from a healthy blood donor and from a patient with gastrointestinal carcinoma. Lanes 1–5, the MAb DH1-reactive EDAcFN; lanes 6–9, BF12-reactive total FN. Lane 1, a purified standard cFN; lanes 2 and 6, serum of the carcinoma patient; lanes 3 and 7, plasma from the carcinoma patient; lanes 4 and 8, plasma from a healthy blood donor; lanes 5 and 9, serum from a healthy blood donor.

concentrations of EDAcFN in plasma samples. Normal serum content of EDAcFN was hardly visible. Consistent with EIA results. Western transfer examination revealed an increased concentration of EDAcFN in plasma and serum of a cancer patient compared with healthy blood donors (Figure 4).

### Discussion

Numerous studies have evaluated whether FN levels in plasma or other body fluids might be indicative of cancer or other clinical conditions. The results have been contradictory. High plasma levels of total FN have been reported in patients with breast, ovarian, lung, pancreatic and colonic carcinoma (Mosher and Williams, 1978; Parsons *et al*., 1979*a*,*b*; Todd *et al*., 1980; Choate and Mosher, 1983), whereas reduced levels of FN have been reported in plasma of patients with lymphatic leukaemia (Bruhn and Heimburger, 1976) and haemopathic diseases (De Russe *et al*., 1985). Interestingly, the latter diseases differ from carcinomas in that they lack a stromal response. On the other hand, Eijan *et al*. (1986) found no clear correlation between FN levels and breast cancer, and normal FN concentrations have also been reported in patients with acute leukaemia, lacking the stromal response (Choate and Mosher, 1983). Moreover, the FN level often increases in various benign conditions (Todd *et al*., 1980). Accordingly, the clinical usefulness of total plasma FN as a marker for cancer seems limited. Recently, immunoassays measuring different fragments or isoforms of FN have been evaluated in the diagnosis of benign and malignant diseases. Katayama *et al*. (1991) have studied FN fragments in urine as a tumour marker. Different isoforms of FN, such as EDAcFN and oncofetal FN, have so far been used as markers for other clinical conditions such as vascular injury or acute pulmonary injury (Peters *et al*., 1988, 1989) or as a predictor of preterm delivery (Lockwood *et al*., 1991).

Recently, we described an EIA detecting EDAcFN (Ylätupa *et al*., 1993). In this study, the normal values of EDAcFN in plasma and serum were determined by studying blood donors. Low concentrations of EDAcFN were found in both plasma and serum of healthy individuals. The concentration in serum was clearly lower than in plasma, which apparently is due to binding of FN to fibrin in blood clotting (Engvall *et al*., 1978). It has previously been shown that the levels of total FN in plasma increase significantly with age and that there are minor differences in total FN concentrations between men and women (Fyrand and Solumn, 1976; Peters *et al*., 1989). However, the concentrations of EDAcFN in serum and plasma seem to be independent of sex and age. Our observations are in concordance with those of Peters *et al*. (1989).

Immunohistochemically, a strong expression of EDAcFN has been shown in malignant tumours using the monoclonal antibody DH1 against the EDA domain of cFN (Vartio *et al*., 1987; Gould *et al*., 1990). In this study we performed a preliminary evaluation of the potential utility of our new assay as a plasma and/or serum tumour marker by determining the EDAcFN levels in 120 cancer patients. A majority of patients with gastrointestinal cancers showed elevated values in both plasma and serum. The same was true for a small group of miscellaneous tumours, including lung, kidney, liver, bladder and a few other cancers. In spite of the higher concentrations found in plasma, the sensitivity of the test, when the 97.5% level of blood donors were used as a cut-off level, was similar in plasma and serum. In breast cancer, however, there was a difference between plasma and serum. Sixty per cent of the patients had an elevated serum level of EDAcFN, whereas the concentration in plasma was low in most patients. On the other hand, the elevated serum levels in many breast cancer patients were only slightly above the cut-off level, and in 30% of patients with benign diseases the serum level exceeded the cut-off level. In plasma samples from all patients with benign disease the concentration remained below the cut-off level.

Since the levels of various tumour markers are often increased during pregnancy, EDAcFN was measured in 20 plasma samples from pregnant women. The mean concentration was higher than that of healthy blood donors, but remained clearly below the cut-off level of normal subjects.

Theoretically, plasma should be a more reliable medium than serum for determining the level of EDAcFN in the circulation, since the amount of FN lost in blood clotting is unpredictable. Using ROC analysis, EDAcFN in serum seems a more specific marker for breast cancer. However, for malignancies other than breast cancer there was only a minor difference between serum and plasma samples. The number of patients studied is, however, too small for definite conclusions to be drawn.

In conclusion, the results of the new EDAcFN EIA as a tumour marker test are very encouraging, and a larger number of patients with various cancers, different stages of cancer and benign diseases are now being collected for further evaluation.

**Acknowledgements**

We thank Professor Ismo Virtanen's research group and Locus genex Oy for monoclonal antibodies and Professor Ulf-Håkan Stenman for samples from pregnant women. The skilful technical assistance of Dr Tapani Tiusanen and Mr Pekka Hyytiäinen is acknowledged. This study was supported by the Finnish Medical Research Council, the University of Helsinki and Locus genex Oy, Helsinki, Finland.

**References**

BOCCARDO F, GUARNERI S, CASTELLANI P, BORSI L AND ZARDI L. (1986). Fibronectin concentration in the plasma of patients with malignant and benign breast disease. *Cancer Lett.*, **33**, 317–323.

BRUHN HD AND HEIMBURGER N. (1976). Factor VIII-related antigen and cold inducible globulin in leukemia and carcinomas. *Haemostasis*, **5**, 189–192.

CHOATE J AND MOSHER DF. (1983). Fibronectin concentration in plasma of patients with breast cancer, colon cancer and acute leukemia. *Cancer*, **51**, 1142–1147.

DE RUSSE J, COLOMBAT B, LAVOIX X AND BARDOS P. (1985). Plasma fibronectin in various hemopathic diseases. *Clin. Chim. Acta*, **145**, 49–58.

EIJAN AM, PURICELI L, BAL DE KIER JOFFE EB, ENTIN D, VUOTO D, ORLANDO E AND DE LUSTIG ES. (1986). Serial analysis of fibronectin concentration in plasma of patients with benign and malignant breast diseases. *Cancer*, **57**, 1345–1349.

ENGVALL E AND RUOSLAHTI E. (1977). Binding of soluble form of fibroblast surface protein, fibronectin, to collagen. *Int. J. Cancer*, **20**, 1–5.

ENGVALL E, RUOSLAHTI E AND MILLER EJ. (1978). Affinity of fibronectin to collagens of different genetic types and to fibrinogen. *J. Exp. Med.*, **147**, 1584–1595.

FAIRBANKS G, STECK TL AND WALLACH DFH. (1971). Electrophoretic analysis of the major polypeptides of the human erythrocyte membrane. *Biochemistry*, **10**, 2606–2617.

FYRAND O AND SOLUMN NO. (1976). Studies on cold insoluble globulin in dermatological patients. I. Immunochemical quantitation in citrated plasma from patients with increased amounts of heparin precipitable fraction (HPF). *Thromb. Res.*, **9**, 447–455.

GOULD VE, KOUKOULIS GK AND VIRTANEN I. (1990). Extracellular matrix proteins and their receptors in the normal, hyperplastic and neoplastic breast. *Cell. Different. Dev.*, **32**, 409–416.

KATAYAMA M, HINO F, KAMIHAGI K, SEKIGUCHI K, TITANI K AND KATO I. (1991). Urinary fibronectin fragments (a potential tumor marker) measured by immunoenzymometric assay with domain-specific monoclonal antibodies. *Clin. Chem.*, **37**, 466–471.

LAEMMLI UK. (170). Cleavage of structural proteins during the assembly of the head of bacteriophage T4. *Nature*, **227**, 680–685.

LAITINEN L, VARTIO T AND VIRTANEN I. (1991). Cellular fibronectins are differentially expressed in human fetal and adult kidney. *Lab. Invest.*, **64**, 492–498.

LOCKWOOD CJ, SENYEI AE, DISCHE R, CASAL D, SHAH KD, THUNG SN, JONES L, DELIGDISCH L AND GARITE TJ. (1991). Fetal fibronectin in cervical and vaginal secretions as a predictor of preterm delivery. *N. Engl. J. Med.*, **325**, 669–674.

LOWRY OH, ROSEBROUGH NJ, FARR AL AND RANDALL RJ. (1951). Protein measurement with the Folin phenol reagent. *J. Biol. Chem.*, **193**, 265–275.

MOSHER DF. (1980). Fibronectin. *Prog. Hemost. Thromb.*, **5**, 111–151.

MOSHER DF AND WILLIAMS EM. (1978). Fibronectin concentration is decreased in plasma of severely ill patients with disseminated intravascular coagulation. *J. Lab. Clin. Med.*, **91**, 729–735.

PARSONS RG, ALDENDORFER PH AND KOWAL R. (1979*a*). Detection of a human serum DNA-binding protein associated with malignant disease. *J. Natl Cancer Inst.*, **63**, 43–47.

PARSONS RG, TODD HD AND KOWAL R. (1979*b*). Isolation and identification of a human serum fibronectin-like protein elevated during malignant disease. *Cancer Res.*, **39**, 4341–4345.

PETERS JH, GINSBERG MH, CASE CM AND COCHRANE CG. (1988). Release of soluble fibronectin containing an extra type III domain (ED1) during acute pulmonary injury mediated by oxidants of leucocytes *in vivo*. *Am. Rev. Resp. Dis.*, **138**, 167–174.

PETERS JH, MAUNDER RJ, WOOLF AD, COCHRANE CG AND GINSBERG MH. (1989). Elevated plasma levels of ED1+ ('cellular') fibronectin in patients with vascular injury. *J. Lab. Clin. Med.*, **113**, 586–597.

RUELLAND A, KERBRAT P, CLERC C, LEGRAS B AND CLOAREC L. (1988). Level of plasma fibronectin in patients with breast cancer. *Cancer Clin. Chim. Acta*, **178**, 283–287.

SCHWARZBAUER JE. (1991). Fibronectin: from gene to protein. *Curr. Opin. Cell Biol.*, **3**, 781–786.

SIRI A, CARNEMOLLA B, CAFFANTI S, CASTELLANI P, BALZANO E AND ZARDI L. (1984). Fibronectin concentration in pleural effusions of patients with malignant and non-malignant diseases. *Cancer Lett.*, **22**, 1–9.

STATHAKIS NE, FOUNTAS A AND TSIANOS E. (1981). Plasma fibronectin in normal subjects and in various disease states. *J. Clin. Pathol.*, **34**, 504–508.

TAMKUN JW AND HYNES RO. (1983). Plasma fibronectin is synthesized and secreted by hepatocytes. *J. Biol. Chem.*, **258**, 4641–4647.

TODD HD, COFFEE MS, WAALKES TP, ABELOFF MD AND PARSONS RG. (1980). Serum levels of fibronectin and fibronectin-like DNA-binding protein in patients with various diseases. *J. Natl Cancer Inst.*, **65**, 901–904.

TOWBIN H, STAEHELIN T AND GORDON J. (1979). Electrophoretic transfer of proteins from polyacrylamide gels to nitrocellulose sheets: procedure and some applications. *Proc. Natl Acad. Sci. U.S.A.*, **76**, 4350–4354.

VARTIO T, LAITENEN L, NÄRVÄNEN O, CUTOLO M, THORNELL L-E, ZARDI L AND VIRTANEN I. (1987). Differential expression of the ED sequence-containing form of cellular fibronectin in embryonic and adult human tissues. *J. Cell. Sci.*, **88**, 419–430.

VIRTANEN I, LAITINEN L AND VARTIO T. (1988). Differential expression of the extra domain-containing form of cellular fibronectin in human placentas at different stages of maturation. *Histochemistry*, **90**, 25–30.

WEBB KS AND LIN GH. (1980). Urinary fibronectin. Potential as a biomarker in prostatic cancer. *Inv. Urol.*, **17**, 401–404.

YAMADA K, AKIYAMA SK, HASEGAWA T, HASEGAWA E, HUMPHRIES MJ, KENNEDY DW, NAGATA K, URUSHIHARA H, OLDEN K AND CHEN, W-T. (1985). Recent advances on research of fibronectin and other cell attachment proteins. *J. Cell. Biochem.*, **28**, 78–98.

YLÄTUPA S, HAGLUND C, PARTANEN P AND VIRTANEN I. (1993). Competitive enzyme immunoassay for quantitation of cellular form of fibronectin (EDAcFN) in blood samples. *J. Immunol. Methods*, **163**, 41–47.